# HIV, pathology and epigenetic age acceleration in different human tissues

**DOI:** 10.1007/s11357-022-00560-0

**Published:** 2022-04-11

**Authors:** Steve Horvath, David T. S. Lin, Michael S. Kobor, Joseph A. Zoller, Jonathan W. Said, Susan Morgello, Elyse Singer, William H. Yong, Beth D. Jamieson, Andrew J. Levine

**Affiliations:** 1 Department of Human Genetics, David Geffen School of Medicine, University of California Los Angeles, Los Angeles, CA 90095 USA; 2 Department of Biostatistics, Fielding School of Public Health, University of California Los Angeles, Los Angeles, CA 90095 USA; 3 Centre for Molecular Medicine and Therapeutics, BC Childrens Hospital Research Institute, Vancouver, Canada; 4 Department of Pathology and Jonsson Comprehensive Cancer Center, David Geffen School of Medicine, Los Angeles, USA; 5 Department of Neurology, Icahn School of Medicine at Mount Sinai, New York, NY USA; 6 Departments of Neuroscience and Pathology, Icahn School of Medicine at Mount Sinai, New York, NY USA; 7 Department of Neurology, David Geffen School of Medicine, University of California, Los Angeles, USA; 8 Department of Medicine, David Geffen School of Medicine, University of California, Los Angeles, USA

**Keywords:** Epigenetic clock, DNA methylation, Cross tissue analysis, HIV, Hypertension

## Abstract

Epigenetic clocks based on patterns of DNA methylation have great importance in understanding aging and disease; however, there are basic questions to be resolved in their application. It remains unknown whether epigenetic age acceleration (EAA) within an individual shows strong correlation between different primary tissue sites, the extent to which tissue pathology and clinical illness correlate with EAA in the target organ, and if EAA variability across tissues differs according to sex. Considering the outsized role of age-related illness in Human Immunodeficiency Virus-1 (HIV), these questions were pursued in a sample enriched for tissue from HIV-infected individuals. We used a custom methylation array to generate DNA methylation data from 661 samples representing 11 human tissues (adipose, blood, bone marrow, heart, kidney, liver, lung, lymph node, muscle, spleen and pituitary gland) from 133 clinically characterized, deceased individuals, including 75 infected with HIV. We developed a multimorbidity index based on the clinical disease history. Epigenetic age was moderately correlated across tissues. Blood had the greatest number and degree of correlation, most notably with spleen and bone marrow. However, blood did not correlate with epigenetic age of liver. EAA in liver was weakly correlated with EAA in kidney, adipose, lung and bone marrow. Clinically, hypertension was associated with EAA in several tissues, consistent with the multiorgan impacts of this illness. HIV infection was associated with positive age acceleration in kidney and spleen. Male sex was associated with increased epigenetic acceleration in several tissues. Preliminary evidence indicates that amyotrophic lateral sclerosis is associated with positive EAA in muscle tissue. Finally, greater multimorbidity was associated with greater EAA across all tissues. Blood alone will often fail to detect EAA in other tissues. While hypertension is associated with increased EAA in several tissues, many pathologies are associated with organ-specific age acceleration.

**Supplementary Information:**

The online version contains supplementary material available at 10.1007/s11357-022-00560-0.

## INTRODUCTION

Machine learning-based analyses of DNA methylation changes at cytosine residues of cytosine–phosphate–guanine dinucleotides (CpGs) have generated multivariate age predictors, known as epigenetic clocks that use specific CpG methylation levels to estimate chronological age (i.e., DNAmAge) [1–5] and/or mortality risk [6–8]. When studying the relationship between age-related conditions and DNAmAge, it is important to adjust the analysis for chronological age. To arrive at a non-confounded analysis, one can employ age-adjusted measures of DNAmAge, referred to as measures of epigenetic age acceleration (EAA).

The biological relevance of epigenetic measures of age acceleration can be appreciated by the fact that they relate to a host of age-related conditions and diseases [7]. EAA has been linked to conditions such as neuropathology in the elderly [9, 10], Down syndrome [11], Parkinson's disease [12], Werner syndrome [13], physical/cognitive fitness [9], frailty [14] and centenarian status [15]. In addition to being predictive of all-cause mortality, DNAmAge acceleration in blood is associated with the risk of developing certain types of cancer [16–19]. In older individuals, positive EAA in blood is associated with an increased risk of death from all natural causes even after accounting for known risk factors [20–24].

The pan tissue clock [3] is particularly attractive for studying epigenetic aging effects in several different tissues. For example, Down syndrome is associated with strong EAA in both blood and brain tissue [11]. Similarly, we have shown that HIV infection is associated with EAA in both blood and brain tissue [25–28], and that age acceleration in brain is associated with HIV-associated neurocognitive disorder [10]. These results lead to natural questions: 1) does EAA in one tissue (e.g., blood) correlate with EAA in another tissue (e.g., brain), 2) is EAA in an organ associated with tissue pathology and clinical illness, 3) does EAA in specific organs underlie the higher rate of age-related illness among HIV-infected individuals, and 4) as our previous studies have shown that males age faster than females according to an epigenetic clock analysis of blood and brain tissue, are there sex differences in EAA across tissues?

By generating a large methylation data set from 661 postmortem samples derived from 11 types of tissue, this study addresses several outstanding questions surrounding epigenetic clocks. First, to quantify the extent to which EAA in one tissue correlates with EAA in another tissue. We use both human tissues and animal tissues to address this question. Second, to relate measures of human tissue pathology and clinical illness to EAA in the same tissue or organ. Third, to define a multimorbidity index that correlates with EAA in several non-blood tissues. Fourth, to extend previous findings from our group that HIV accelerates aging in blood and brain, by investigating the effect of HIV infection on age acceleration across these tissues. Finally, to study the effect of sex on EAA in different human tissues.(Table 1)

**Table 1:** Age and sex of persons from which tissue samples were derived

Tissue	Total N	Female N	Mean Age	Min. Age	Max. Age
Adipose	57	18	57.3	27.9	77.6
Blood	32	8	55	26.2	76.1
Bone Marrow	20	6	54.7	38.3	73.9
Heart	97	38	56.1	27.9	91.2
Kidney	97	38	55.7	27.9	91.2
Liver	97	40	56.2	27.9	91.2
Lung	113	42	55.7	27.9	85.3
Lymph Node	27	9	60.2	38.3	77.6
Muscle	57	17	56.7	27.9	77.6
Pituitary	2	0	54.3	43.7	64.9
Spleen	62	19	57.3	27.9	85.3

## RESULTS

**Fig. 1:**
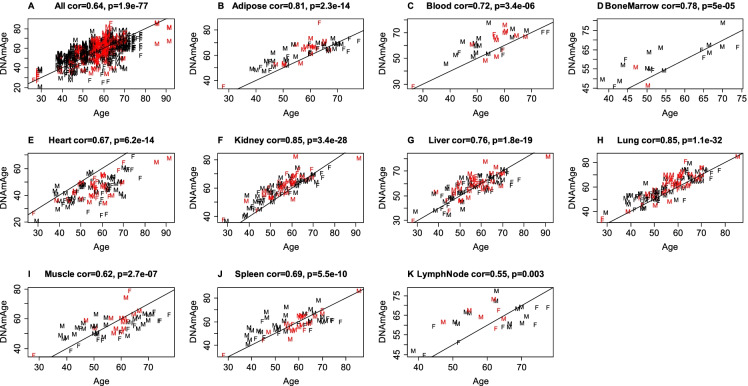
**Pan tissue clock applied to different postmortem tissues**. DNAmAge estimate (y-axis) versus chronological age at the time of sample collection. A) All tissues, B) adipose, C) blood, D) bone marrow E) heart, F) kidney, G) liver, H) lung, I) muscle, J) spleen and K) lymph nodes. Dots are colored by hypertension status (red = diagnosis of hypertension) and labeled by sex (F = female, M = male). The title of each plot reports the Pearson correlation coefficient. The solid line corresponds to the diagonal y = x

The mammalian methylation array platform (HorvathMammalMethylChip40) [29] array allowed us to calculate two different epigenetic clocks: the pan tissue clock^3^ and the skin and blood clock [5]. Since we are working with many different tissues, we primarily focus on the results from the pan tissue clock herein. As expected, the DNAmAge estimate of the pan tissue clock is highly correlated with chronological age across all tissues (r = 0.64, Fig. 1A) and within specific tissues (Fig. 1B-K). The age correlations persisted even with the somewhat limited age range of our sample, which was skewed towards older individuals (median age 57, range 26 to 91).

## Conservation of EAA across different human tissues

**Fig. 2:**
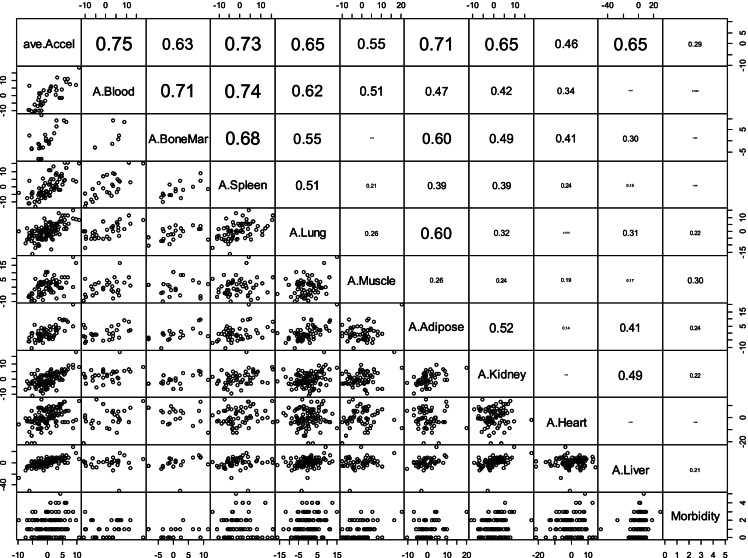
**Conservation of EAA across different human tissues**. The diagonal reports the respective variables for each row: EAA measures and multimorbidity index. The panels below the diagonal show the pairwise scatter plots. The numbers above the diagonal report the corresponding Pearson correlation coefficients between listed tissue for the row and the tissue listed lower down in the column. Each dot corresponds to a different person. For example, the multimorbidity index (last column) is correlated with epigenetic age acceleration in muscle (Pearson correlation 0.30). The measures of EAA were calculated within each tissue type based on the pan tissue clock. A.Blood denotes the epigenetic age acceleration in blood, i.e., the age adjusted measure of DNAmAge in blood tissue. The first variable, ave.Accel, denotes the average EAA across all tissues. Average EAA per individual, ave.Accel in the first column, was defined as average EAA across the following measures of EAA: A.Adipose, A.Blood, A.BoneMar, A.Heart, A.Kidney, A.Liver, A.Lung, A.LymphNode, A.Muscle and A.Spleen. Morbidity denotes the multimorbidity index. The analysis was limited in that each comparison involved a different set of individuals due to missing values

EAA is defined as the residual resulting from regressing DNAmAge on chronological age within the respective tissue. As illustrated in Fig. 2, we found that EAA in human blood is highly correlated with EAA in spleen (r = 0.74), bone marrow (r = 0.71), lung (r = 0.62), muscle (r = 0.51), adipose (r = 0.47), kidney (r = 0.42) and heart (r = 0.34), but not in liver tissue. EAA in liver was instead correlated with EAA in kidney (r = 0.49), adipose (r = 0.41), lung (r = 0.31) and bone marrow (r = 0.30).

## Tissue pathology and clinical illness

We present a detailed analysis of all measures of tissue pathology and clinical diagnoses versus EAA in lung (Supplementary Fig. 7), liver (Supplementary Fig. 8), heart (Supplementary Fig. 9) and kidney (Supplementary Fig. 10).

**Fig. 3:**
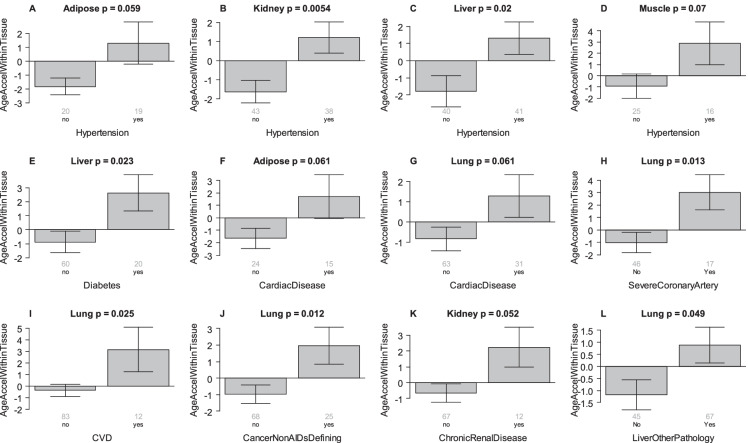
**Age-related conditions versus EAA in different tissues.** Epigenetic age acceleration (y-axis) versus different conditions (x-axis) in different human tissues. A) Adipose, B) Kidney, C) Liver, D) Muscle, E) Liver, F) Adipose, G-J) Kung, K) Kidney. L) Lung. We caution the reader that liver-other-pathology (panel L) is not a clinical disease and may not be age related. A-D) relates hypertension status to epigenetic age acceleration. I) CVD denotes cerebrovascular disease. J) Non-AIDS defining cancer status

We also found significant associations between EAA in different tissues and hypertension, diabetes, cardiac disease, severe coronary artery disease, cerebrovascular disease, non-AIDS defining cancers, chronic renal disease and liver disease (e.g., hepatitis or end stage liver disease) (Fig. 3). Of particular interest, hypertension is associated with EAA, according to the pan tissue clock^3^, across all tissues combined (p = 4.5E-5, Supplementary Fig. 2A), as well as individually with kidney (p = 0.0048, Supplementary Fig. 2F), liver (p = 0.028, Supplementary Fig. 2G) and lymph nodes (p = 0.033, Supplementary Fig. 2 K). According to the skin and blood epigenetic clock^5^, hypertension was again associated with EAA across all tissues combined (p = 0.00036, Supplementary Fig. 3A), and individually in kidney (p = 0.0095, Supplementary Fig. 3F).

## Multimorbidity

For our study, we defined a novel multimorbidity index as the number of the following conditions per person: hypertension, type II diabetes, cardiovascular disease, non-AIDS defining cancer, chronic renal disease and hepatitis. For example, an individual with three conditions (e.g., hypertension, diabetes and cardiovascular disease) was assigned a multimorbidity index score of 3. Descriptive statistics surrounding the multimorbidity index, age, HIV status and sex are presented in Supplementary Fig. 5. By design, the multimorbidity index is positively correlated with EAA in different tissues (Supplementary Fig. 6). Nominally significant positive correlations (p < 0.05) were observed in kidney, liver, lung and muscle (Supplementary Fig. 6D-G).

Multivariable regression models that included age and sex revealed that the multimorbidity index is significantly associated with average EAA across all tissues (p = 5.26E-4, Table 2). However, the average EAA, age and sex explain only 17% of the variance in the multimorbidity index (17%, R^2 = 0.17). We also evaluate the relationship between multimorbidity and DNAmAge in specific tissues (Table 2). Strikingly, the pan tissue clock showed a nominally significant association (two-sided p < 0.062) with the multimorbidity index after correcting for age and sex.

**Table 2:** Multivariable regression model of the multimorbidity index

Model 1. Outcome = Morbidity Index, Overall R-squared: = 0.17				
**Covariate**	**Coef**	**Std.Error**	**T statistic**	**P-value**
Age	0.0283	0.0087	3.26	1.42E-03
ave.Accel	0.0880	0.0247	3.56	5.26E-04
Female	0.3764	0.2046	1.84	6.82E-02
**Model 2. Kidney, Outcome = Morbidity Index**				
Age	-0.0193	0.0166	-1.16	2.48E-01
DNAmAgeKidney	0.0510	0.0202	2.52	1.30E-02
Female	0.2814	0.1883	1.49	1.38E-01
**Model 3. Liver, Outcome = Morbidity Index**				
Age	-0.0015	0.0136	-0.11	9.15E-01
DNAmAgLiver	0.0299	0.0126	2.36	2.00E-02
Female	0.1428	0.2167	0.66	5.11E-01
**Model 4. Lung, Outcome = Morbidity Index**				
Age	-0.0026	0.0167	-0.15	8.79E-01
DNAmAgeLung	0.0481	0.0178	2.70	7.84E-03
Female	0.1041	0.2059	0.51	6.14E-01
**Model 5. Muscle, Outcome = Morbidity Index**				
Age	-0.0093	0.0122	-0.76	4.48E-01
DNAmAgeMuscle	0.0454	0.0181	2.51	1.45E-02
Female	0.0361	0.2322	0.16	8.77E-01
**Model 6. Adipose, Outcome = Morbidity Index**				
Age	-0.0247	0.0194	-1.27	2.08E-01
DNAmAgeAdipose	0.0438	0.0230	1.90	6.17E-02
Female	-0.1637	0.2523	-0.65	5.19E-01
**Model 7. Lymph Node, Outcome = Morbidity Index**				
Age	-0.0267	0.0232	-1.15	2.61E-01
DNAmAgeLymph	0.0679	0.0335	2.03	5.36E-02
Female	0.2402	0.4476	0.54	5.96E-01

The dependent variable (multimorbidity index) was regressed on chronological age, female status (sex) and DNAmAge calculated using the pan tissue clock. DNAmAge is equivalent to AgeAccel in a multivariate regression model that includes chronological age as covariate. The table presents the results from seven different multivariable regression models that differ with respect to the DNAm-based biomarker under investigation. Model 1 presents the results of the average EAA across all considered tissues. Average EAA per individual, ave.Accel, was defined as average EAA across the following measures of EAA: A.Adipose, A.Blood, A.BoneMar, A.Heart, A.Kidney, A.Liver, A.Lung, A.LymphN, A.Muscle, A.Spleen. Missing values were omitted from the average. Models 2–7 present analogous results in kidney, liver, lung, skeletal muscle tissue, adipose and lymph nodes, respectively. The table reports all results whose nominal p value for the methylation-based covariate was significant with a nominal p value less than 0.10

The multimorbidity index correlated with average EAA across all tissues (r = 0.29, Fig. 2) and EAA in muscle (r = 0.30), adipose (r = 0.24), lung (r = 0.22), kidney (r = 0.22) and liver (r = 0.21) but not blood. We caution the reader that our analysis involving the multimorbidity index is biased since the conditions underlying the index were chosen so that the resulting index exhibits a positive association with EAA. Another source of bias arises from uneven missingness patterns. Figure 2 may be biased because each pairwise comparison uses different people due to missing values. Therefore, we repeated the analysis limited to 77 individuals with at most 2 missing values across a subset of tissues. We found qualitatively the same results (Supplementary Fig. 4).

## HIV status and ALS status

A scatter plot reveals that kidney tissue from HIV-positive individuals exhibit increased DNAmAge compared to that of HIV-negative individuals (Supplementary Fig. 1). Multivariable regression model analysis finds that HIV status is positively associated with DNAmAge in kidney (p = 1.66E-4) but negatively associated with DNAmAge in muscle (p = 1.16E-4) even after adjusting for the morbidity index and sex (Table 3).

**Table 3:** Multivariable regression model of HIV status

Covariate	Coef	Std. Error	T-stat	P value
**Model 1: Average Epigenetic Accel**				
HIVstatus	-0.1007	0.7923	-0.13	8.99E-01
Age	0.0231	0.0329	0.70	4.84E-01
Female	-1.8369	0.7247	-2.53	1.25E-02
**Model 2: Kidney DNAmAge**				
HIVstatus	3.6646	0.9623	3.81	2.30E-04
Age	0.7486	0.0402	18.62	6.10E-36
Female	-0.2140	0.8423	-0.25	8.00E-01
**Model 3. Muscle DNAmAge**				
HIVstatus	-5.7095	1.4722	-3.88	2.32E-04
Age	0.3945	0.0554	7.12	7.04E-10
Female	-5.9968	1.2966	-4.63	1.64E-05
**Model 4: Average Epigenetic Accel**				
**Covariate**	**Coef**	**Std. Error**	**T-stat**	**P value**
HIVstatus	0.4268	0.7720	0.55	5.81E-01
Age	-0.0017	0.0322	-0.05	9.57E-01
Female	-1.9792	0.6944	-2.85	5.10E-03
Morbidity	1.0534	0.2936	3.59	4.74E-04
**Model 5: Kidney DNAmAge**				
HIVstatus	3.6537	0.9370	3.90	1.66E-04
Age	0.7310	0.0397	18.42	2.13E-35
Female	-0.4712	0.8257	-0.57	5.69E-01
Morbidity	1.0575	0.3971	2.66	8.91E-03
**Model 6. Muscle DNAmAge**				
HIVstatus	-5.7410	1.4060	-4.08	1.16E-04
Age	0.3734	0.0534	6.99	1.32E-09
Female	-5.6548	1.2443	-4.54	2.24E-05
Morbidity	1.8116	0.6468	2.80	6.59E-03
**Model 7: Spleen DNAmAge**				
HIVstatus	3.3242	1.8866	1.76	8.23E-02
Age	0.5758	0.0699	8.24	5.45E-12
Morbidity	0.3798	0.6895	0.55	5.83E-01

The table presents the results of different multivariable regression models that differ with respect to the DNAm-based biomarker under investigation. ave.Accel = average EAA across multiple tissues. Model 1 presents the results of the average EAA across all considered tissues. Models 2–4 present results for DNAm age in kidney, skeletal muscle and spleen, respectively. The table reports only reports the regression results the case where the methylation-based dependent variable led an association test result with the morbidity index whose nominal p value was less than 0.10

Since the negative association between HIV and DNAmAge in muscle was unexpected, we carried out two follow-up analyses. First, we investigated whether testosterone treatment taken by HIV-positive individuals could explain this unexpected negative association. We did not find a significant association between testosterone treatment and DNAmAge in muscle among the 20 HIV positive men for whom testosterone treatment status was available at the time of death (9 treated and 11 untreated). Second, we studied whether confounding by amyotrophic lateral sclerosis (ALS) could explain the unexpected negative association. Our study involved 18 ALS cases (5 HIV positive and 13 HIV negative ALS cases). ALS was associated with significantly increased DNAmAge (5.5 years, p = 0.0038) in muscle tissue even after adjusting for age and sex. However, neither ALS nor HIV status was significantly associated with DNAmAge in muscle after including both covariates in a multivariate regression model along with sex. This insignificant effect of both covariates may be due to significant multi-collinearity (chi-square test p = 5.1 × 10^–7^) between ALS and HIV status: most ALS cases did not have HIV and vice versa. Overall, the negative association between DNAmAge and HIV status in muscle tissue is no longer significant (p > 0.2) after we adjust for ALS status.

When using the skin and blood clock^5^ for the spleen samples, we found that HIV status is associated with epigenetic age acceleration (multivariate regression model Wald test p = 0.02 in a model that only included Age and HIV status). The latter result echoes our original finding that HIV accelerates epigenetic age of blood since epigenetic aging effects are highly correlated between spleen and blood (r = 0.74, Fig. 2).

## Effect of sex on EAA across tissues/organs

**Fig. 4:**
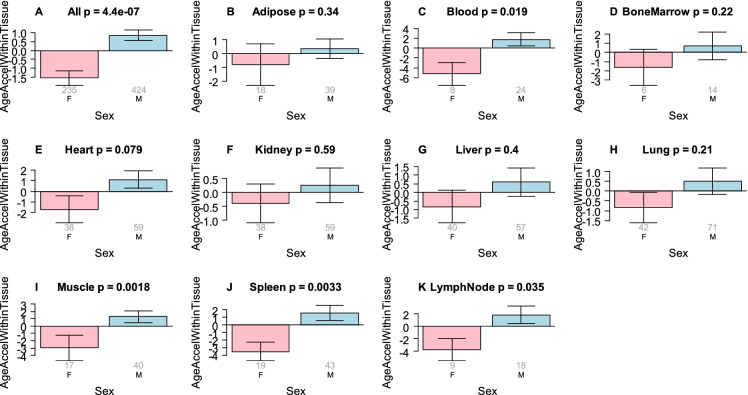
**Effect of sex on EAA in different tissues**. EAA within tissue (y-axis) is defined as the residual resulting from regressing DNAmAge on chronological age within a given tissue type. The grey numbers underneath each bar report the number of samples from females and males (x-axis). The title of each panel reports the result from a non-parametric group comparison test (Kruskal–Wallis test). A) Results for all tissues combined, i.e., the analysis ignores tissue type. B-K report the results for different tissues/organs B) adipose, C) blood, D) bone marrow, E) heart, F) Kidney, G) liver, H) lung, I) muscle, J) spleen, K) lymph node

Men were found to exhibit higher EAA than women across all tissues when analyzed together (p = 4.4E-7, Fig. 4A). Statistically significant results were also observed in individual tissues (muscle, spleen and lymph nodes; Fig. 4I-K).

## Conservation of EAA across different baboon tissues

Since our cross tissue analysis of epigenetic age acceleration in humans may be confounded by long postmortem intervals, we repeated the analysis for two animal models. Our pan tissue clock for baboons (Papio hamadryas) [30] revealed weak pairwise correlations between epigenetic age acceleration across different baboon tissues (Supplementary Fig. 11). The strongest correlations could observed between baboon cerebral cortex and cerebellum (r = 0.44), cerebral cortex and heart (r = 0.36), cerebral cortex and muscle (r = 0.30) and adipose and liver (r = 0.40).

## Conservation of EAA across different swine tissues

We used leave one sample out estimates of epigenetic age acceleration based on the pan tissue clock for pigs [31]. We restricted the analysis to pigs for whom all 6 tissues were available (bladder, blood, frontal cortex, kidney, liver and lung), i.e., the same number of animals were studied for each tissue. We found moderate pairwise correlations between epigenetic age acceleration of the pan tissue clock across different pig tissues. Relatively strong pairwise correlations could be observed for epigenetic age acceleration in liver tissue which was positively correlated with epigenetic age acceleration in kidney (r = 0.62), lung (r = 0.48), frontal cortex (r = 0.45), blood (r = 0.44) and bladder (r = 0.25).

Epigenetic age acceleration in lung was correlated with epigenetic age acceleration kidney (r = 0.79), blood (r = 0.48), and liver r = 0.48) but not with frontal cortex (r = -0.09).

Epigenetic age acceleration in pig blood was correlated with epigenetic age acceleration in kidney (r = 0.43), liver (r = 0.44) and lung (r = 0.48), but it only related very weakly with frontal cortex (r = 0.14) and bladder (r = 0.18).

## DISCUSSION

In a previous study, we showed that sex was associated with EAA in blood and brain tissue. The current study extends this finding to many other tissues [32]. We initiated this investigation with the benefit of experience gained from our work with epigenetic clocks. [3, 5, 6, 8] As expected, we found that epigenetic age is largely correlated across tissues and organs. Blood had the greatest number and degree of correlations, most notably with spleen and bone marrow. Epigenetic age in blood did not correlate with that of liver. Instead, EAA in liver was weakly correlated with EAA in kidney, adipose, lung and bone marrow. In general, heart, liver and muscle had the weakest and fewest correlations with other tissues and organs. These findings indicate that 1) blood remains the best candidate for measuring overall EAA, and 2) the epigenetic age of tissues and organs accelerates at different rates, with some more independent than others.

In a previous study, we only found weak (but significant) positive correlations (correlation between 0.04 and 0.07) between systolic blood pressure and EAA in blood [33]. Therefore, we were surprised to learn that hypertension was associated with EAA in several organs and tissues (heart, kidney, liver and muscle) according to the pan tissue clock. This could simply reflect the fact that hypertension leads to pathology in these organs [34]. Notably, another group recently reported that accelerated aging according to alternative epigenetic clocks is associated with organ damage in kidney, heart, brain and peripheral arteries [35]. However, these effects were attenuated when clinical factors (BMI, diabetes and smoking history) were included in the analysis. Conversely, inclusion of hypertension in the analyses generally did not attenuate the significance of the associations between epigenetic age and tissue pathology. Similar to our previous findings, the other research group did not find their epigenetic aging measures to be associated with diastolic or systolic blood pressure.

By design, our multimorbidity index, which is quantified as the sum of up to six medical conditions, is positively correlated with EAA in liver, lung, muscle and kidney, as well as a composite measure of age acceleration across all tissues sampled. Future studies, in which pre-mortem medical conditions are thoroughly documented, might consider applying alternative multimorbidity indexes, such as those that provide weights for more serious medical conditions [36, 37] and which have been applied in specific contexts, such as HIV research. [38–40]

Several studies have examined the effect of HIV on methylation levels in different organs [27, 41, 42].

Compared to tissues from uninfected individuals, those from HIV-positive individuals exhibit increased DNAmAge in kidney. HIV-1 infection is associated with an increased risk for a number of diseases and medical conditions typically associated with aging, including cardiovascular disease, osteoporosis, several cancers, kidney disease, liver disease and cognitive decline [43–55]. We previously demonstrated the clinical relevance of DNAm-based accelerated aging in HIV-infected individuals. Specifically, we reported that the brains of deceased adults diagnosed with neurocognitive impairment within a year of death had greater age acceleration than those who were cognitively normal [56]. More recently, we reported that neurodevelopment and neuropsychological deficits in perinatally HIV-infected adolescents are associated with accelerated aging [26, 42]. The current findings support age acceleration only in kidney, as well as an association between age acceleration and kidney disease. However, the lack of findings of relatively greater age acceleration in other tissues does not support the hypothesis that HIV-induced accelerated aging leads to increased incidence of age-related medical conditions via direct effects on underlying organs.

The unexpected finding that HIV status is negatively associated with DNAmAge in muscle (even after adjusting for the morbidity index and sex) probably reflects confounding by ALS status. In the same data, we found that ALS was associated with significant positive age acceleration in muscle tissue. The nature of ALS disease causes muscle atrophy as the nerve no longer innervates the muscle. Strictly speaking, strong confounding/multi-collinearity between ALS status and HIV status does not allow us to distinguish between the following possibilities: either ALS is associated with positive age acceleration or HIV is associated with negative age acceleration in muscle. The majority of our HIV + patients were men and either worked out and/or may have received anabolic steroids as therapy. Future studies focusing on these aspects will be needed to resolve the observed complexity.

Overall, this study reveals only moderate conservation of epigenetic aging effects across different tissues from 3 different species: human, baboon and pig. Overall, we expect that blood will often be a suboptimal surrogate for other tissues when it comes to epigenetic aging effects. This judgement is based on a) the moderate correlation coefficients between age acceleration in blood and that of other tissues and b) the fact that several conditions are associated with tissue specific age acceleration effects. It will be advisable to profile several sources of DNA (including blood, buccal cells, adipose and skin) to get a comprehensive picture of the epigenetic aging state of an individual.

## METHODS

Tissue Samples: This study was conducted in accordance with the University of California, Los Angeles Medical Institutional Review Board (IRB). Clinical and pathological data and biological samples came from 133 HIV-infected and uninfected individuals enrolled in either the National Neurological AIDS Bank (NNAB) or Manhattan HIV Brain Bank MHBB) sites of the National NeuroAIDS Tissue Consortium (NNTC)^57,58^. All individuals died between 2001 and 2016. These biorepositories operate in accordance with their local IRBs and act as “honest brokers” in maintaining participant confidentiality. All samples were obtained with approved consents allowing for genetic analysis. Donors contributing to this study died between 2001 and 2016.

Tissue Pathology: At the time of autopsy, representative samples of all tissues were obtained and flash frozen, as well as fixed in formalin. Formalin fixed tissues were processed for paraffin embedding and routine histology. Hematoxylin and eosin stains were examined by board certified anatomic pathologists (SM and JS), and special stains obtained as indicated by the histopathology. Slides were reviewed by two pathologists to arrive at concordant diagnoses.

Clinical Characterization: Age and sex of donors for each tissue are displayed in Table 1. Medical diagnoses were obtained by self-report and/or medical record review. The NNTC routinely collects data on hypertension, diabetes, dyslipidemia, hepatitis and liver disease, chronic renal disease, cardiac disease, chronic obstructive pulmonary disease, cancer, cerebrovascular disease and diverse neurologic conditions.

Multimorbidity: The conditions underlying the multimorbidity index were chosen so that the resulting index would exhibit a positive association between the index and EAA. In other words, the analysis is biased. Thus, the p-value should be interpreted as descriptive measure as opposed to an inferential measure. Our version of the multimorbidity index counts the number of conditions an individual had at the time of death. The selected conditions were: hypertension, type-2 diabetes, cardiovascular disease (CVD), non-AIDS defining cancers, chronic renal disease and hepatitis.

DNA Methylation: We used the mammalian methylation array ^29^ to generate DNA methylation data from n = 672 samples representing 11 human tissues (adipose, blood, bone marrow, heart, kidney, liver, lung, lymph node, muscle, spleen and pituitary gland). We removed n = 11 samples from the analysis since they were outliers according to hierarchical clustering, the inter-array correlation coefficient, or detection p values.

All methylation data were generated using a custom Illumina methylation array (HorvathMammalMethylChip40) based on 37,492 CpG sites ^29^. Of these, 1986 CpGs were cgiseb based on their utility for human biomarker studies; these CpGs, which were previously implemented in human Illumina Infinium arrays (EPIC, 450 K, 27 K), were selected due to their relevance for estimating human age, human blood cell counts or the proportion of neurons in human brain tissue. The particular subset of species for each probe is provided in the chip manifest file at the NCBI Gene Expression Omnibus (GEO) platform (GPL28271). The "noob" normalization method was used to define beta values using the *minfi* R package ^59,60^.

## Supplementary Information

Below is the link to the electronic supplementary material.

Supplementary file1 (DOCX 541 kb)
