# Supplementary material for: HIV, pathology and epigenetic age acceleration in different human tissues
Source: GeroScience. 2022 Apr 11;44(3):1609–20. doi: 10.1007/s11357-022-00560-0 (PMC9213580; doi:10.1007/s11357-022-00560-0)
Supplement: Supplementary file 1 — Supplementary file1 (DOCX 541 kb) [file 11357_2022_560_MOESM1_ESM.docx]

**Supplementary Material**

**Supplementary Figure 1. Detailed analysis of lung tissue.** Epigenetic age acceleration (y-axis) versus various conditions (x-axis). Each barplot reports a two sided Kruskal Wallis test p value. Each bar depicts mean values and one standard error. Group sizes (counts) are reported under each bar.

Epigenetic age acceleration (y-axis) versus A) abnormal plumonary system status, B) diffuse alverolar damaage, C) pulmonary thromboembolus, D) chronic pulmonary disease, E) lung cancer, F) other pathology.

**Supplementary Figure 2. Detailed analysis of liver.** Epigenetic age acceleration (y-axis) versus various conditions (x-axis). Each barplot reports a two sided Kruskal Wallis test p value. Each bar depicts mean values and one standard error. Group sizes (counts) are reported under each bar.

**Supplementary Figure 3. Detailed analysis of aorta and heart.** Epigenetic age acceleration in the heart (y-axis) versus various conditions (x-axis). Some of the conditions pertain to the aorta (e.g. atherosclerosis in panels B,C). Panel E is a subset of those who have a Yes value in panel D. Each barplot reports a two sided Kruskal Wallis test p value. Each bar depicts mean values and one standard error. Group sizes (counts) are reported under each bar.

**Supplementary Figure 4. Detailed analysis of heart.** A) X axis labels 0 and 1 refer to no and yes respectively. Epigenetic age acceleration in kidney (y-axis) versus various conditions (x-axis). Each barplot reports a two sided Kruskal Wallis test p value. Each bar depicts mean values and one standard error. Group sizes (counts) are reported under each bar.

**Supplementary Figure 5. Hypertension status versus EAA of the pan tissue clock.** EAA was defined as residual resulting from regressing DNAmAge on chronological age within the respective tissue. Hypertension status (x-axis) versus EAA in A) all tissues, B) adipose, C) blood, D) bone marrow E) heart, F) kidney, G) liver, H) lung, I) muscle, J) spleen, K) lymph nodes. The title of each panel reports a Kruskal Wallis test p-value. The bar plots report the mean value and one standard error. The small grey numbers under each bar report group sizes.

**Supplementary Figure 6. Hypertension versus EAA according to the skin and blood clock**. EAA based on the pan tissue clock from Horvath 2018 (y-axis) versus hypertension status (x-axis) in A) all tissues, B) adipose, C) blood, D) bone marrow E) heart, F) kidney, G) liver, H) lung, I) muscle, J) spleen, K) lymph nodes. The title of each panel reports a Kruskal Wallis test p-value. The bar plots report the mean value and one standard error. The small grey numbers under each bar report group sizes. EAA was defined as residual resulting from regressing DNAmAge on chronological age within the respective tissue.

**Supplementary Figure 7. Descriptive statistics surrounding the multimorbidity index versus, age, HIV status, and sex**. A) Histogram of the multimorbidity index. B) Histogram of age, C) Multimorbidity index versus age, D) Proportion of females versus HIV grouping status. Multimorbidity index versus E) HIV status, and F) female status (1=female). We caution the reader that these marginal associations are confounded. For example, the relationship between the morbidity index and HIV status is confounded by age and sex. For this reason, we use multivariable regression models.

**Supplementary Figure 8. Multimorbidity index versus EAA in different tissues.**

Each panel reports the tissue/organ, the Pearson correlation coefficient and corresponding p value.

**Supplementary Figure 9. Conservation of EAA across different tissues limited to individuals with few missing values**. This is the analog of Figure 3 but it involves only n=77 individuals with at most 2 missing value per measure of EAA. The diagonal reports the respective variables: EAA measures and multimorbidity index. The panels below the diagonal show the pairwise scatter plots. The numbers above the diagonal report the corresponding Pearson correlation coefficients. Each dot corresponds to a different person. The measures of EAA were calculated within each tissue type based on the pan tissue clock (Horvath 2013). The first variable, ave.Accel, denotes the average EAA across all tissues. Average EAA per individual, ave.Accel, was defined as average EAA across the following measures of EAA: A.Adipose, A.Blood, A.BoneMar, A.Heart, A.Kidney, A.Liver, A.Lung, A.LymphN, A.Muscle, A.Spleen. Here A.Blood denotes EAA in blood. Morbidity denotes the multimorbidity index.

**Supplementary Figure 10. Pan tissue clock applied to different tissues where points are colored by HIV status**.

This figure is similar to Figure 1 but dots are colored by HIV status. The blood data were omitted since they involved only a single HIV positive individual.

**
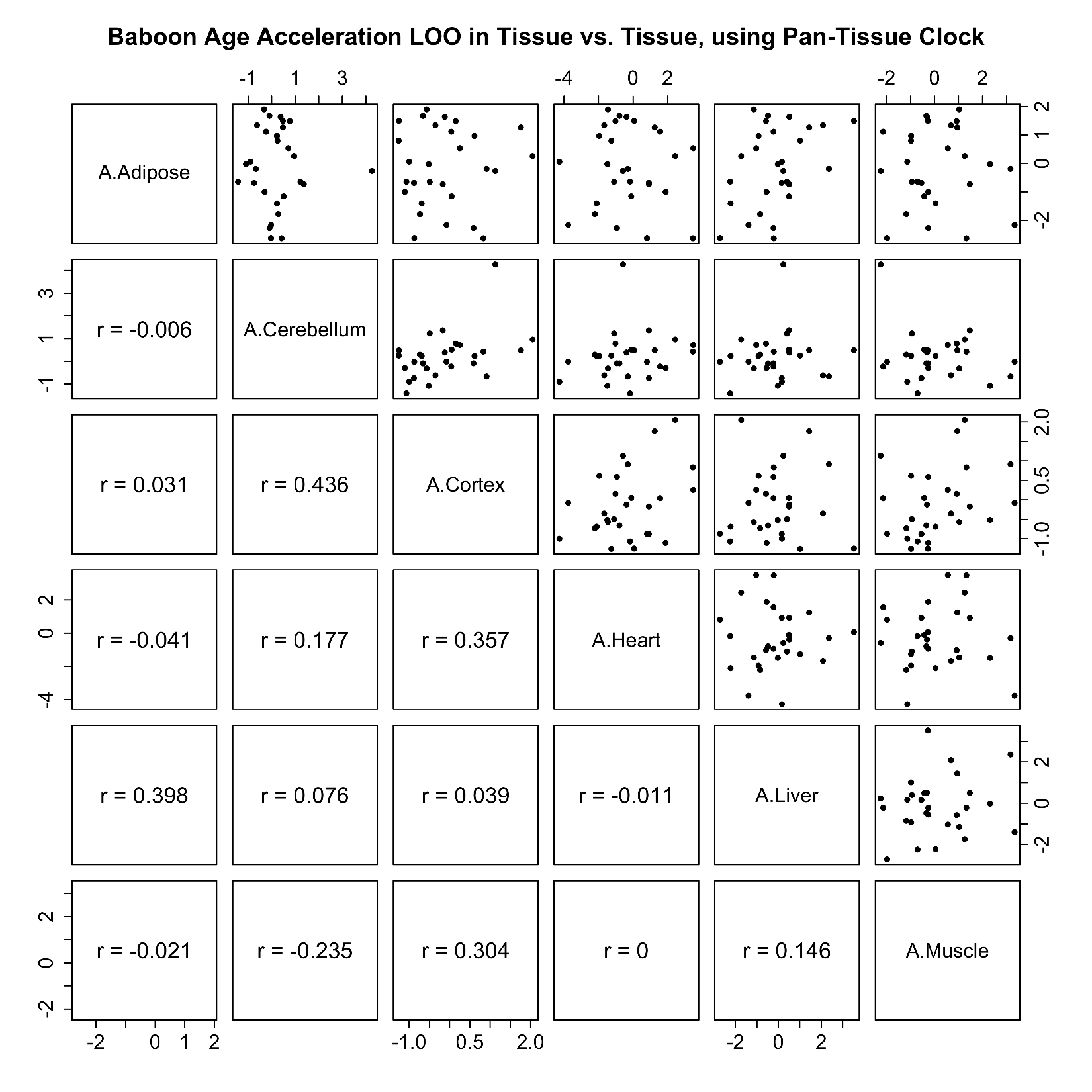
**

**Supplementary Figure 11. Conservation of epigenetic age acceleration across baboon tissues**. The diagonal reports the respective variables for each row: epigenetic age acceleration in different tissues. The panels above the diagonal show the pairwise scatter plots. The numbers below the diagonal report the corresponding Pearson correlation coefficients between listed tissue for the row and the tissue listed lower down in the column. Each dot corresponds to a different animal. The measures of EAA were calculated within each tissue type based on the pan tissue clock for baboons ^1^. We used leave one sample out estimates (LOO) of epigenetic age acceleration based on the baboon pan tissue clock. A.Adipose denotes the LOO estimate of epigenetic age acceleration in adipose, i.e. the age adjusted measure of DNAmAge in adipose tissue. We restricted the analysis to baboons for whom all 6 tissues were available (adipose, cerebellum, cerebral cortex, heart, liver, skeletal muscle), i.e. the same number of animals were studied for each tissue.


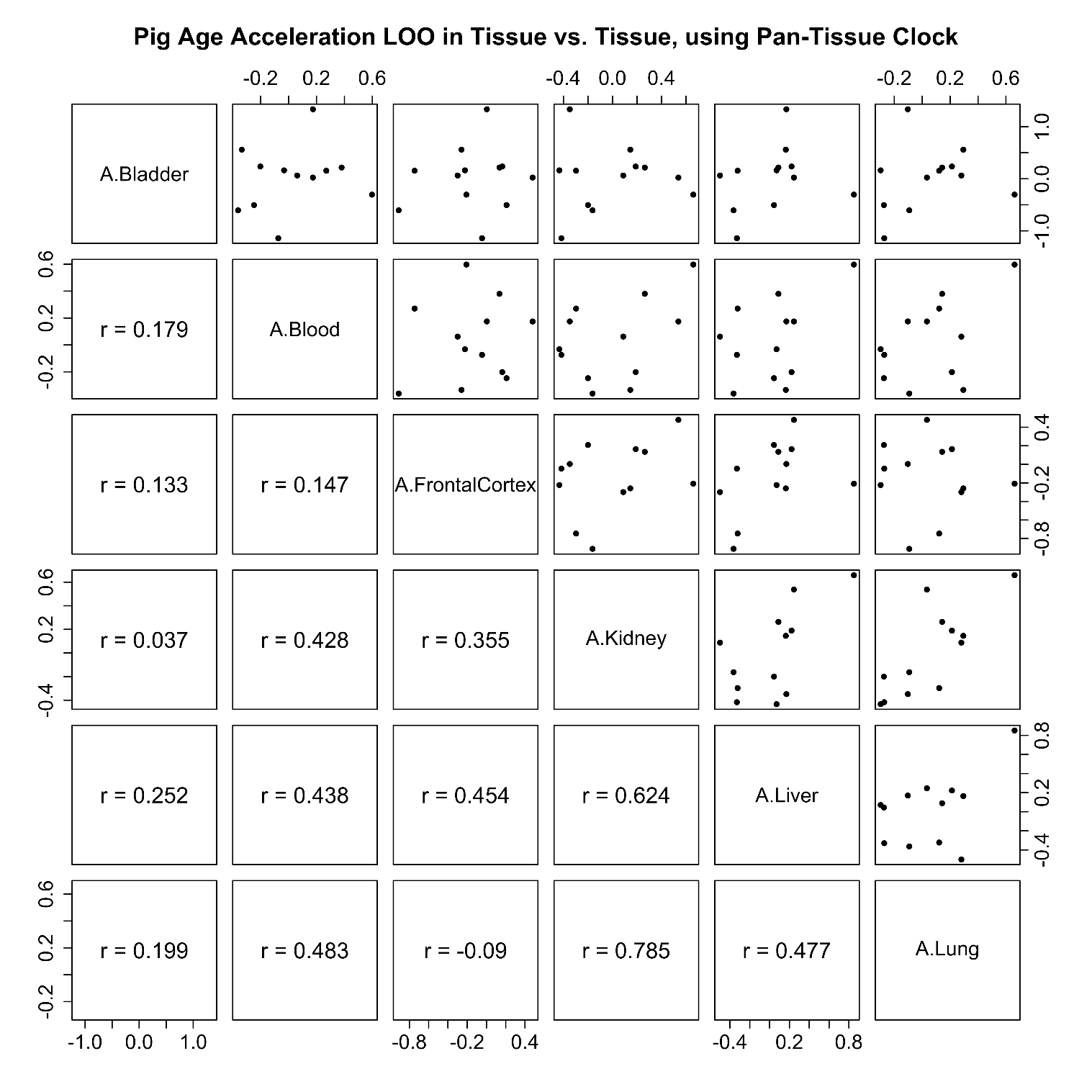


**Supplementary Figure 12. Conservation of epigenetic age acceleration across swine tissues**. The diagonal reports the respective variables for each row: epigenetic age acceleration in different swine tissues. The panels above the diagonal show the pairwise scatter plots. The numbers below the diagonal report the corresponding Pearson correlation coefficients between listed tissue for the row and the tissue listed lower down in the column. Each dot corresponds to a different animal. The measures of EAA were calculated within each tissue type based on the pan tissue clock for pigs ^2^. We used leave one sample out estimates (LOO) of epigenetic age acceleration based on the swine pan tissue clock. We restricted the analysis to pigs for whom all six tissues were available ((bladder, blood, frontal cortex, kidney, liver, lung), i.e. the same number of animals were studied for each tissue.

## REFERENCE

1 Horvath, S. *et al.* Pan-primate DNA methylation clocks. *bioRxiv*, 2020.2011.2029.402891, doi:10.1101/2020.11.29.402891 (2021).

2 Schachtschneider, K. M. *et al.* Epigenetic clock and DNA methylation analysis of porcine models of aging and obesity. *GeroScience*, doi:10.1007/s11357-021-00439-6 (2021).
